# The breakthrough of oxide pathway mechanism in stability and scaling relationship for water oxidation

**DOI:** 10.1093/nsr/nwae362

**Published:** 2024-10-15

**Authors:** Zhao-Hua Yin, Hong Liu, Jin-Song Hu, Jian-Jun Wang

**Affiliations:** State Key Laboratory of Crystal Materials, School of Cystal Materials, Shandong University, Jinan 250100, China; State Key Laboratory of Crystal Materials, School of Cystal Materials, Shandong University, Jinan 250100, China; Beijing National Laboratory for Molecular Sciences (BNLMS), CAS Key Laboratory of Molecular Nanostructure and Nanotechnology, Institute of Chemistry, Chinese Academy of Science, Beijing 100190, China; State Key Laboratory of Crystal Materials, School of Cystal Materials, Shandong University, Jinan 250100, China

**Keywords:** electrocatalyst, oxygen evolution reaction, oxide pathway mechanism, active site, scaling relationship

## Abstract

An in-depth understanding of electrocatalytic mechanisms is essential for advancing electrocatalysts for the oxygen evolution reaction (OER). The emerging oxide pathway mechanism (OPM) streamlines direct O–O radical coupling, circumventing the formation of oxygen vacancy defects featured in the lattice oxygen mechanism (LOM) and bypassing additional reaction intermediates (*OOH) inherent to the adsorbate evolution mechanism (AEM). With only *O and *OH as intermediates, OPM-driven electrocatalysts stand out for their ability to disrupt traditional scaling relationships while ensuring stability. This review compiles the latest significant advances in OPM-based electrocatalysis, detailing design principles, synthetic methods, and sophisticated techniques to identify active sites and pathways. We conclude with prospective challenges and opportunities for OPM-driven electrocatalysts, aiming to advance the field into a new era by overcoming traditional constraints.

## Teaser

Revolutionizing water oxidation: The Oxide Pathway Mechanism discovers the stability and scaling secrets and redefines stability and efficiency in electrocatalysis, guiding future innovation and performance breakthroughs.

## INTRODUCTION

The oxygen evolution reaction (OER) is a cornerstone in the field of electrochemical energy conversion and storage, playing a pivotal role in a variety of sustainable energy technologies [1]. Central to processes such as water electrolysis, metal-air batteries, and photoelectrochemical water splitting, the OER is integral to the production of hydrogen, a clean and renewable fuel, and the rechargeability of energy storage systems [2]. However, the OER is kinetically sluggish and requires significant overpotentials to proceed at practical rates, which leads to substantial energy losses and reduced overall system efficiency [3]. This inefficiency is primarily due to the complex multi-electron transfer process, the formation of strong O–O bonds, and the necessity for the adsorption and desorption of intermediates at the electrode surface [4]. Consequently, the development of efficient and robust electrocatalysts that can lower the overpotential and accelerate the reaction kinetics is a critical challenge that researchers face in maximizing the potential of electrochemical energy systems [5].

To enhance the efficiency of the OER, several important challenges must be tackled. First, the development of catalyst materials with superior intrinsic activity is critical [6,7]; these materials should facilitate the reaction at reduced overpotentials by possessing optimal electronic structures that enable efficient electron transfer and minimize energy barriers for reaction intermediates [8]. Second, the stability of catalysts under the rigorous oxidative conditions of the OER is a paramount concern [2,9]. Catalyst degradation, resulting from corrosion, structural collapse, or surface passivation, is often observed during prolonged operation [10–12]. Thus, it is imperative to enhance the durability of OER catalysts without sacrificing their activity for viable practical applications [13]. Another challenge lies in designing catalysts with surface properties that favor the adsorption of reactants and the desorption of oxygen gas [14]. The surface structure and composition are critical in dictating the reaction mechanism and the pathway of intermediate evolution. Additionally, advanced characterization techniques and theoretical models are indispensable for a profound understanding of the OER mechanism at atomic and molecular levels, which is essential for the rational design and performance optimization of catalysts [15]. Furthermore, the scalability and economic feasibility of OER catalysts are crucial for their widespread implementation. Many high-performance catalysts depend on precious metals like iridium and ruthenium, which are rare and costly [11]. It is therefore urgent to investigate and develop alternative materials, such as abundant and affordable transition metal oxides, hydroxides, and sulfides, with comparable catalytic properties [11,16]. Overcoming these challenges associated with the OER is key to enabling efficient, sustainable, and cost-effective energy systems that are vital for addressing increasing energy demands and reducing environmental impacts.

**Scheme 1.**
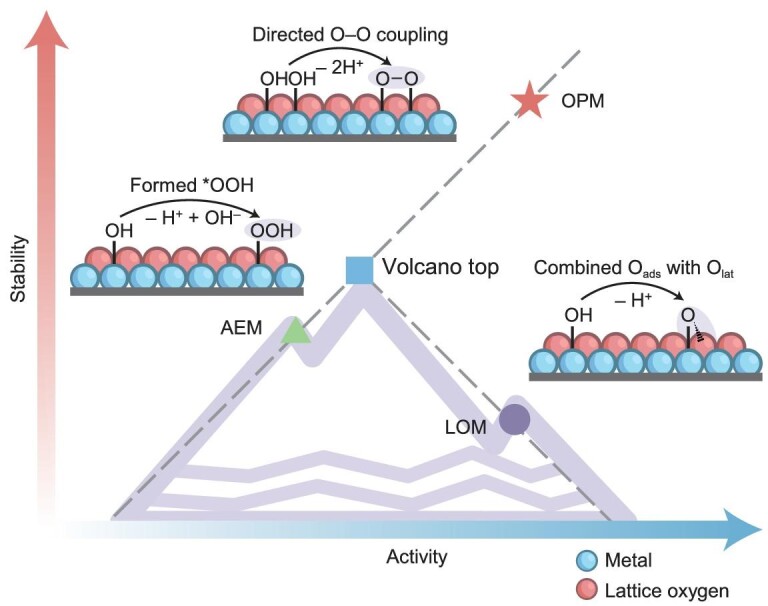
Schematic illustration of the OPM-driven electrocatalysts for OER promising to break the see-saw relationship between activity and stability compared to AEM and LOM.

The exploration of electrocatalytic oxygen evolution mechanisms is a dynamic and critical field that tackles fundamental aspects of energy conversion and storage. Delving into the intricacies of OER mechanisms is of paramount importance for identifying and developing superior electrocatalysts that could revolutionize energy storage and conversion technologies [17]. The conventional adsorbate evolution mechanism (AEM) for OER features metal sites acting as redox centers, undergoing a sequence of four synchronized proton–electron transfer steps: *OH → *O → *OOH → O_2_, where * denotes the active site (Scheme 1) [18]. The kinetics of the reaction are heavily influenced by the binding affinity of these oxygen intermediates at the active sites [19]. A defining characteristic of AEM is the universal scaling law, which correlates the binding energies of *OOH and *OH, expressed as ΔG_*OOH_ = ΔG_*OH_ + 3.2 ± 0.2 eV [20]. This scaling law implies that the binding energies of intermediates are linked and cannot be independently optimized, creating an ‘overpotential wall’ at ∼370 mV, above which substantial improvements in catalytic efficiency are challenging to realize [21]. Despite these limitations, the well-established framework and predictability of AEM have facilitated the systematic optimization of catalyst materials. Transition metal oxides and their derivatives have been thoroughly investigated within this context, laying a robust foundation for future advancements [13,22]. The simplicity of AEM also renders it more accessible for modeling and comprehension. However, the interconnected nature of the intermediate binding energies restricts flexibility in catalyst design and makes it difficult to significantly lower overpotentials [23]. The formation of the *OOH intermediate, often the rate-determining step (RDS), represents a significant hurdle in enhancing reaction kinetics [18]. This challenge, together with the inflexible scaling relationships, constitutes a significant obstacle to surpassing current performance benchmarks. Moreover, despite the general assumption of the stability for the catalytic surface in AEM-based electrocatalysts, the irreversible cation overoxidation leads to the leaching of metal active sites, followed by catalyst dissolution and failure, which has persistently acted as a constraint on achieving new stability records [24].

The lattice oxygen mechanism (LOM), which has recently been extensively studied, diverges significantly from AEM by involving the redox chemistry of lattice oxygen (O_lat_) and non-concerted proton–electron transfer steps [25]. This mechanism involves the participation of lattice oxygen species, traversing the pathway through OH^−^, O_2_^2−^, and O_2_, and includes direct (O–O) bond coupling [18,26]. Activation of lattice oxygen requires elevating the O 2p band center near the Fermi level and intensifying orbital hybridization between the metal d band and the O p band, facilitating intramolecular electron transfer from oxygen ligands to metal cations [27]. This process generates positively charged lattice O^(2−δ)−^ species, which are crucial for active oxygen ligand liberation [28]. Early models of LOM, such as the single lattice oxygen mechanism (SLOM), suggested that an activated lattice oxygen atom (O_lat_) and an adsorbed oxygen atom (O_ads_) combine to form O_ads_O_lat_, which then releases O_2_ [11,27,29]. In contrast, the dual lattice oxygen mechanism (DLOM), elucidated by Hu *et al.*, involves two activated lattice oxygen atoms forming a peroxide intermediate, with dioxygen release from the superoxide intermediate being the RDS [30]. Recent advances, such as the work by Wang *et al.* with Ir_1_@NiOOH catalysts, demonstrate a shift from SLOM to DLOM, achieving significant efficiency improvements [31]. LOM offers the advantage of potentially lower overpotentials and improved catalytic performance by sidestepping the rigid scaling relationships inherent in AEM, offering a potential route to circumvent the ‘overpotential wall’ [23,32]. This adaptability opens up avenues for more creative catalyst designs and the potential to achieve heightened efficiencies. Nonetheless, the participation of lattice oxygen in LOM may induce structural instability due to the sluggish replenishment rate of OH^−^ relative to the rate of oxygen evolution [18]. This discrepancy can lead to the relaxation of oxygen vacancy defects and subsequent deterioration of the electrocatalytic structure and performance, a phenomenon referred to as oxygen anion overoxidation [24]. In essence, the structural degradation of the AEM-based and LOM-based electrocatalysts can be distilled down to the irreversible overoxidation of cations and anions, respectively. Hence, striking a balance between activity and stability remains a pivotal challenge, necessitating novel strategies to preserve catalyst integrity while pushing the boundaries of performance [33].

Steered by the strengths and limitations of both AEM and LOM, there is a strong impetus to develop next-generation OER catalysts that meet the demands of both activity and stability, thereby paving the way for more efficient and stable electrochemical energy systems. In the natural photosystem II, water oxidation is efficiently catalyzed by active structures such as Mn_4_Ca clusters [34]. These clusters feature multinuclear arrangements with two water activation centers at an optimal separation, enabling intramolecular O–O bond formation through what is known as the oxo–oxo coupling mechanism (OCM) in homogeneous catalysis [35,36]. Yet, while homogeneous systems lend themselves to precise, molecular-level design based on mechanistic insights, heterogeneous systems are generally more practical for real-world applications [35].

Recently, the oxide pathway mechanism (OPM) has emerged as an innovative concept in the realm of heterogeneous electrocatalysis for OER. Echoing the principles of OCM, OPM also revolves around the creation of dual active sites, facilitating the independent tuning of the binding energies of intermediates. This approach holds the potential for a significant leap forward, circumventing the traditional scaling relationship that has long-constrained advancements in OER catalysis. At the core of OPM is the process where two adjacent metal centers undergo *OH deprotonation at precisely controlled distances, leading to the formation of two metal-oxygen species [37]. These species can then directly couple, subsequently associating to release an O_2_ molecule [38]. This direct coupling of *O and *OH intermediates, bypassing the formation of the *OOH intermediate typical in AEM, marks a fundamental departure from conventional OER pathways [39]. Additionally, the independent optimization of binding energies at dual active centers in OPM offers a unique opportunity to finely tune catalytic activity [40]. Besides, the OPM avoids the involvement of lattice oxygen, thereby preventing the formation of substantial oxygen vacancy defects that could compromise the stability of the catalyst structure, inherently offering greater stability than mechanisms that involve lattice oxygen [41]. Intriguingly, the OPM naturally precludes cationic overoxidation by addressing significant electronic modulation within the spatial domain between dual atoms and leading to more stable cationic sites compared to those in the AEM mechanism [10,38,42,43]. For instance, the electron transfer from Ce^3+^ within the Ce–O–Ru oxygen bridge effectively inhibits the overoxidation and dissolution of Ru, thus consistently steering the OPM mechanism [44]. Furthermore, the effective OPM-based electrocatalysts are usually derived from an initial unstable structure to a stable state under the applied potential, enabling the resultant OPM-based catalysts to sustain high activity over prolonged periods [45]. Consequently, the OPM adeptly sidesteps the conventional challenges of cation and anion overoxidation encountered in AEM and LOM, preserving the catalytic structure to consistently promote the direct coupling of adsorbed oxygen species for the OER process.

These promising prospects of OPM-driven catalysts provide a significant advantage in harmonizing the often-competing demands of activity and stability within OER catalysis. For instance, a recent seminal study demonstrated an impressive electrocatalytic performance nearing 1.8 A cm^−2^ at 1.77 V for proton exchange membrane water electrolysis (PEMWE) under industrial conditions (80°C) in an acidic medium via the OPM mechanism on a non-noble metal electrocatalyst, cobalt tungstate, which notably maintained stable operation at 1 A cm^−2^ for over 600 hours [37]. By providing a route to simultaneously achieve high catalytic activity and sustained durability, OPM signals the advent of a transformative phase in the advancement of electrocatalysts for energy conversion and storage applications. However, research on OPM-driven catalysts is still in its early stages, and the synthetic challenges surmounted to achieve each reported composition and structure for OPM have resulted in disparate structures in terms of elements and synthesis protocols. Therefore, a systematic understanding of the physicochemical principles underlying OPM is crucial for the design and implementation of OPM-driven catalysts to make significant contributions to the field of sustainable energy.

Herein, we summarize recent advances in the design of optimal electrocatalysts based on OPM. From the concept of OPM, we discuss the design criteria, synthetic strategies, and methodologies for the identification of true active sites and reaction pathways, including *in situ/operando* monitoring techniques and theoretical computational science. Finally, we envisage the remaining challenges and prospects of OPM. We hope that this review can shed light on a new paradigm for researchers to design next-generation catalysts for various applications constrained by OER efficiency.

## DESIGN CRITERIA FOR OPM-DRIVEN ELECTROCATALYSTS

Within the OPM mechanism, two sequences are generally acknowledged based on the evolution of intermediates: (a) one proceeds via OH(I)*–* to O*–* to O*–OH* to O*–O* and finally to *–*, while (b) the other sequence is OH(I)*–* to OH(I)*–OH(II)* to O*–OH* to O*–O* and culminates in *–* [41]. In this review, we concentrate on the mechanisms of O–O bond formation and the origins of the oxygen atoms, without specifically differentiating between these two sequences. While the OPM mechanism exhibits remarkable superiority, it presents more intricate demands on electrocatalyst design, particularly concerning stringent diatomic symmetrical spatial arrangements and electronic configurations that facilitate suitable adsorption energies for oxygen intermediates [20,42,46]. Nonetheless, this also offers fundamental principles for electrode design predicated on the OPM approach.

### Spatial configuration: symmetric dual metal sites with proper atomic spacing

**Figure 1.**
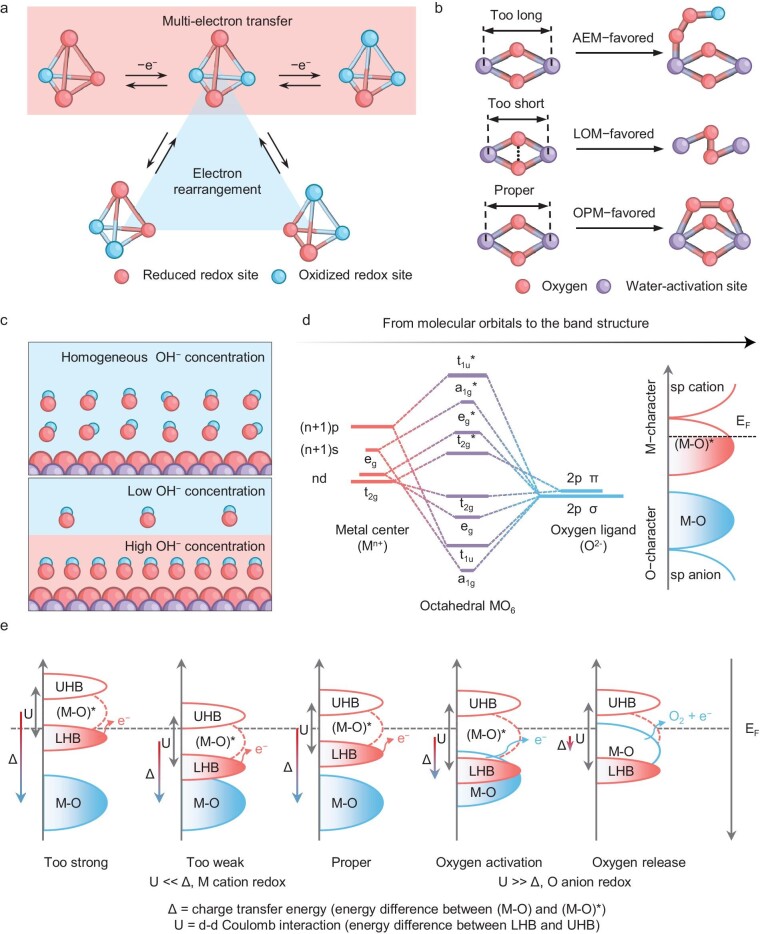
Design criteria for OPM-driven electrocatalysts. (a) Redox flexibility arising from a multinuclear core. Adapted with permission [35]. Copyright 2016, Springer Nature. (b) The spatial configuration of the reaction path fit. (c) OH^−^-rich local pH microenvironments for OPM. (d) The schematic band structure of octahedral-symmetric TMO_6_ in TMOs under the rationalization of molecular orbital theory. Adapted with permission [4]. Copyright 2021, Royal Society of Chemistry. (e) The quest for an ideal electronic configuration of OPM-driven OER electrocatalysts based on cation/anion redox chemistry with the d–d Coulomb interaction (U) and charge transfer energy (Δ) as steering.

The paranormal synergistic effects of bimetallic active sites heavily rely on specific spatial arrangements, including the positioning of metal sites and the distance between metals [20,40,47]. Considering electronic orbital interactions, symmetric bimetallic sites with optimal atomic spacing ensure the redox flexibility of the system, facilitating multi-electron transfer reactions and promoting O–O radical couplings with low energy barriers (Fig. 1a) [35,48]. Generally, pristine oxide structures with elongated M–M spacing are unfavorable for the direct coupling of O–O radicals, directing the reaction pathway towards AEM (Fig. 1b, top) [49]. Recent studies have extensively reported that a reduced M–M distance favors the direct coupling of O–O radicals [20,41,42]. However, excessively short M–M distances may lead to shortened bond lengths between the metal and lattice oxygens, enhancing their orbital hybridization and triggering the LOM (Fig. 1b, middle) [50]. Hence, a moderate expansion of the metal-lattice oxygen bond angle is necessary to accommodate a sufficiently elongated metal-lattice oxygen bond length. Therefore, we propose that the ideal M–O_lat_–M configuration conducive to OPM should strike a balance between the pristine oxide structure (favorable for AEM) and the LOM-preferred structure (Fig. 1b, bottom). Reflecting on the wealth of research conducted on OPM-based electrocatalysts, it is plausible to assert that the optimal interatomic distance between metal active sites conducive to initiating the OPM pathway generally falls within the range of 2.4 Å to 2.9 Å [1,9,17,20,38,41,51,52]. Yet, considering the potential variations in the optimal M–O_lat_–M configurations across diverse crystal structures and multivariate atomic systems, the theoretical calculation could prove instrumental in predicting the specific optimal value for a given configuration beforehand.

### Electronic configuration: balancing OH binding and stabilizing lattice oxygen

The OPM operates by engaging *O and *OH intermediates, deliberately circumventing the formation of *OOH species. The reaction rate for OPM electrocatalysts is often attributed to the slow replenishment of OH^−^ and the sluggish evolution or desorption of oxygen intermediates at transition metal sites [41,42]. In addition, Zhao *et al.* reported on the pH sensitivity of O–O bond formation pathways during photoelectrochemical water oxidation [53,54]. They noted a shift from the AEM to the OPM as a result of increased local pH at elevated photocurrents, which led to accelerated water oxidation kinetics without the accumulation of stable intermediates. This finding suggests that the adsorption and surface coverage of reaction intermediates not only influence the water oxidation rate but can also fundamentally change the reaction mechanism. This concept aligns with observations in other critical reactions such as CO_2_ reduction and nitrate reduction [55–58]. From a relatively macroscopic perspective, such effects can be modulated by engineering local electric fields [59] or creating Lewis acid–base pairs [60]. At a more fundamental level, harnessing the OPM pathway necessitates a thorough understanding of the electronic structure at the active site to enhance the local OH^−^ concentration (Fig. 1c) and to fine-tune the binding properties of oxygen intermediates, thus achieving balanced OH energetics [41,61]. Moreover, to effectively channel the reaction through the OPM pathway, it is crucial to inhibit the activation and participation of lattice oxygen.

The orbital interactions in OER electrocatalysts, as exemplified by the TMO_6_ octahedral structure, can be understood through molecular orbital theory. The overlap between the oxygen 2p orbitals (O 2p) and the transition metal nd orbitals (TM nd) creates bonding (M–O) and antibonding (M–O)* bands, reflecting oxygen and metal features, respectively, abbreviated as O 2p bands and TM nd energy bands (Fig. 1d) [4,62,63]. The charge transfer energy (Δ) represents the energy difference between these bands and serves as an indicator of the extent of orbital hybridization; lower Δ values suggest stronger hybridization [62]. Moreover, due to pronounced d–d Coulomb interactions, the antibonding state (M–O)* is further split into the electron-occupied lower Hubbard band (LHB) and the empty upper Hubbard band (UHB), separated by an energy gap denoted by the parameter U [64]. These parameters, U and Δ, are instrumental in characterizing the redox properties of the system, particularly the redox activities of oxygen anions and metal cations [4,18,29]. In instances where U is considerably less than Δ (U << Δ), the O 2p band is located beneath the LHB, and the conduction gap is primarily governed by d–d interactions. Under these conditions, electrons are drawn from the LHB for the OER, with the metal cation serving as the adsorption and redox site for reaction intermediates [25]. The interaction strength between oxygen intermediates and the active site is often represented by a volcano plot, indicating that optimal adsorption is achieved when the antibonding state is proximate to the Fermi energy level, which implies a desirable state of electronic half-filling [65–67]. Deviations in adsorption strength can lead to either overly strong or weak adsorption (Fig. 1e) [68]. Conversely, when U is substantially greater than Δ (U >> Δ), the O 2p band is positioned above the LHB, and the conduction gap is defined by charge-transfer interactions. Here, electrons are derived from the O 2p band of the activated lattice oxygen, not the LHB, with the oxygen anion acting as the adsorption and redox site [18]. Elevating the O 2p band to align closely with the Fermi level makes the lattice oxygen more likely to be released from the lattice matrix [69]. Shao *et al.* have identified that an optimal charge-transfer energy (∼1 eV) coupled with an ideal electron cloud density near the Fermi level yields the highest catalytic activity, in line with Sabatier's principle [70]. Pristine oxides with a charge-transfer energy of ∼1 eV exhibit metal-like high-valence states and a relatively dense O 2p electron cloud around the Fermi level, contributing to proton adsorption in the hydrogen evolution reaction (HER) and facilitating lattice-oxygen involvement in the OER, respectively. Therefore, the OER pathway can be tuned by fine-tuning U and Δ to manage both the absolute energy of the O 2p band and its relative positioning to the TM nd band. In summary, the effective OPM-based electrocatalysts must fulfill the following electronic structural criteria: (1) the antibonding state (M–O)* should be aligned close to the Fermi level to ensure moderate adsorption strength for intermediates; and (2) the condition U << Δ should be preserved, designating the metal as the primary adsorption and redox site, thereby stabilizing the metal-lattice oxygen interface.

## STRATEGIES FOR DEVELOPING OPM-DRIVEN ELECTROCATALYSTS

**Figure 2.**
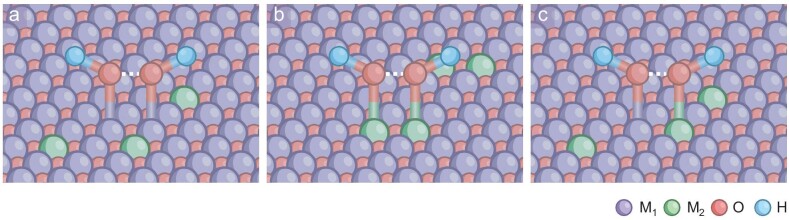
The types of diatomic configurations. (a, b) M_1_–M_1_ and M_2_–M_2_ homonuclear dual-atom sites. (c) M_1_–M_2_ heteronuclear dual-atom sites.

Introducing the dopant element M_2_ into the host crystal structure (M_1_ matrix) can intentionally adjust the active site to achieve desired geometric and electronic characteristics [71]. This leads to a substantial synergistic effect between the two neighboring metal atoms, which in turn fine-tunes the electronic structure of the active site and the adsorbate's binding dynamics, thereby steering the reaction towards the OPM pathway. Depending on the combination of metal atoms at the catalytic center, the diatomic configurations can be classified as homonuclear (including M_1_–M_1_ and M_2_–M_2_) and heteronuclear (M_1_–M_2_) dual-atom sites (Fig. 2) [43]. In this section, we encapsulate how cutting-edge research activates and consistently sustains the OPM mechanism throughout the OER process.

### Homonuclear dual-atom sites configuration

Introducing the dopant element M_2_ into the M_1_ host matrix can optimize the chemical environment at the M_1_ site, modulate charge distribution, create structural irregularities and lattice strain, and even induce phase changes, which collectively promote the transition of M_1_–M_1_ sites to a state more favorable for OPM initiation. Here, M_1_–M_1_ serves as an intermediate adsorption and redox site, while M_2_ functions as a ligating element (Fig. 2a) [48].

**Figure 3.**
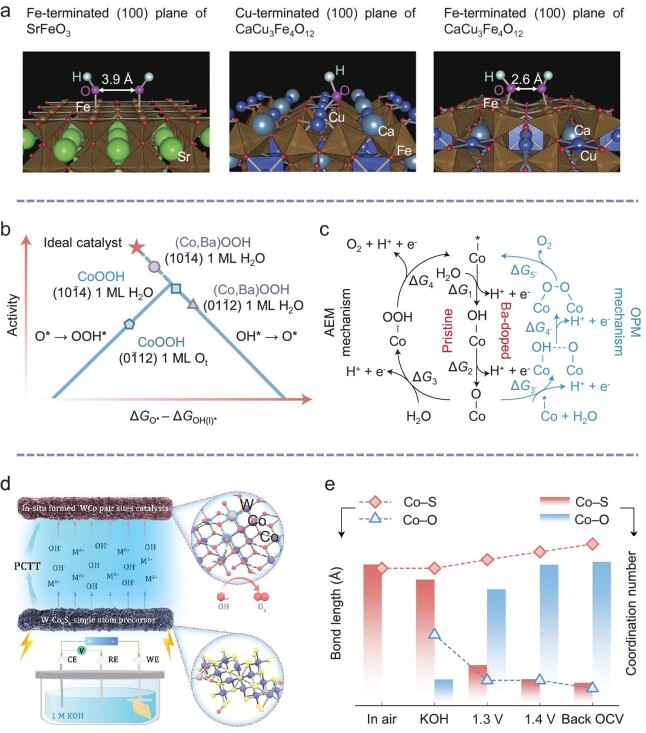
The catalytic center with M_1_–M_1_ configuration. (a) OH^−^ adsorbed surfaces for SFO and CCFO. Left: OH^−^ adsorbates on the Fe-terminated (100) plane of SFO for the Fe-mediated route (AEM). Middle: OH^−^ adsorbate on (Ca, Cu)O-terminated (100) planes of CCFO for the Cu-mediated route (AEM). Right: OH^−^ adsorbates on FeO_2_-terminated (100) planes of CCFO for Fe-mediated route (OPM). Adapted with permission [49]. Copyright 2015, Springer Nature. (b) OER volcano plot showing the predicted theoretical overpotential (*η*_theory_, V) versus the free energy difference between the formation of O* and OH* (Δ*G*_O*_ − Δ*G*_OH(I)*_, eV). Δ*G*_OH(I)*_ denotes the free energy for the formation of the first OH* in the OPM mechanism, whereas it is for OH* formation in the AEM. (c) OPM (Ba-doped) vs AEM (pristine) OER mechanism for catalysts in acidic electrolyte. Adapted with permission from [41]. Copyright 2023, American Chemical Society. (d) Topochemical transformation process from the single-atom catalyst precursor to the W-CoOOH-TT pair-site catalysts by potential-cycling self-reconstruction, where WCo represents W-CoOOH-TT, and (e) *in situ* XAFS tracks topochemical transitions during potential-driven cycling. Adapted with permission [1]. Copyright 2024, Wiley-VCH GmbH.

Yagi and colleagues have synthesized a covalency-strengthened Fe^4+^-based quadruple perovskite, CaCu_3_Fe_4_O_12_ (CCFO), depicted in Fig. 3a [49]. In the conventional cubic ABO_3_-type perovskite structure SrFeO_3_ (SFO), the shortest interatomic distance between neighboring OH^−^ adsorbates is ∼3.9 Å, due to the linear Fe–O–Fe bond angle of 180°, which is conducive only to the AEM pathway. By contrast, CCFO crystallizes in a cubic quadruple AA'_3_B_4_O_12_-type structure, where the A-sites are filled with alkaline earth metal Ca ions, A'-sites with Jahn–Teller active Cu ions, and B-sites with d-block transition metal Fe ions. The significant electronic interactions between A'-Cu and B-Fe ions lead to a covalent bonding network involving multiple Cu^2+^ and Fe^4+^ ions, which effectively reduces the oxygen–oxygen distance to ∼2.6 Å at the acutely angled Fe–O–Fe bond (around 140°). This promotes the direct coupling of adjacent oxygen atoms adsorbed at the Fe site, rather than those at Ca or Sr sites. Sargent *et al.* have introduced Ba ligand cations into the Co_3_O_4_ lattice to synthesize Co_3−_*_x_*Ba*_x_*O_4_, resulting in decreased Co–Co distances and stronger OH^−^ adsorption (Fig. 3b, c) [41]. Consequently, the reaction pathway was switched from AEM in CoOOH to OPM in BaCoOOH. An unprecedented low overpotential of 0.14 V was achieved on the (10–14) facet with a monolayer of H_2_O on (Co, Ba)OOH, defying the traditional volcano plot that correlates theoretical overpotential with the Δ*G*_O*_ − Δ*G*_OH(I)*_ relationship. Additionally, Zr-doped Co_9_S_8_/Co_3_O_4_ heterostructures (Zr-Co_9_S_8_/Co_3_O_4_) were engineered, exhibiting strong electronic interaction between Zr atoms and their substrate [51]. These structures favor dinuclear Co sites with optimal Co–Co spacing (∼2.8 Å) that promote direct O–O bonding. Remarkably, the Zr incorporation generates S–Co–O heterogeneous interfaces with elongated Co–O bonds, leading to electron redistribution that finely controls the adsorption and release of oxygen intermediates. This culminates in the ideal spatial and electronic conditions that activate the OPM, delivering outstanding acidic OER performance (155 mV vs RHE at 10 mA cm^−2^) and maintaining stable operation over 500 hours at 100 mA cm^−2^.

Dynamic reconstruction during the OER process is widely observed in both alkaline and acidic environments [72]. This phenomenon entails significant changes in spatial and electronic configurations [73]. By understanding and harnessing these dynamic transformations, we can refine the design of ideal OPM-based electrocatalysts. Concurrently, single-atom catalysts, with their well-defined active sites and high atom utilization efficiencies, offer exceptional catalytic activity and selectivity [31,74]. Each atom, whether serving as an active site or a modifying ligand, is accessible on the catalyst surface. The strong metal–support interaction (SMSI) between the dispersed single atoms and their support has recently garnered significant interest [45,51]. This interaction is pivotal in altering the chemisorption characteristics, interfacial structure, and electronic properties of the adsorbed metal, challenging the preconceived notion that single-atom catalysts are less stable than their bulk-phase counterparts. Sun and colleagues have focused on leveraging the tuning effects of single-atom ligands for OER dynamic reconstruction, exploring OPM mechanisms [1]. They transformed single-atom catalysts into M-CoOOH-TT (M = W, Mo, Mn, V, etc.) pair-site catalysts with a linear Co–Co–W arrangement through an electrochemical cycling topochemical transformation strategy (Fig. 3d). *In situ* XAFS investigations revealed a potential-driven topological transition in alkaline media, influenced by W ligands. This transition is marked by an irreversible phase shift from Co_9_S_8_ to CoOOH, with elongated Co–S bond lengths and reduced coordination numbers, as well as contracted Co–O bond lengths and increased coordination numbers under applied bias (Fig. 3e). Additionally, the significant contribution from coordination shells between 2.40 and 2.45 Å primarily pertains to the di–µ–oxo bridged Co ions. The restructured W-CoOOH-TT pair-site catalyst, with an optimal Co–Co active site distance, enhances the conversion of oxygenates through oxygen bridge formation. This is particularly effective for *OH binding at the dual Co site, subsequent deprotonation, and the facilitation of oxygen–oxygen coupling leading to O–O bond formation, indicating a two-site OER mechanism rather than the traditional single-site AEM.

**Figure 4.**
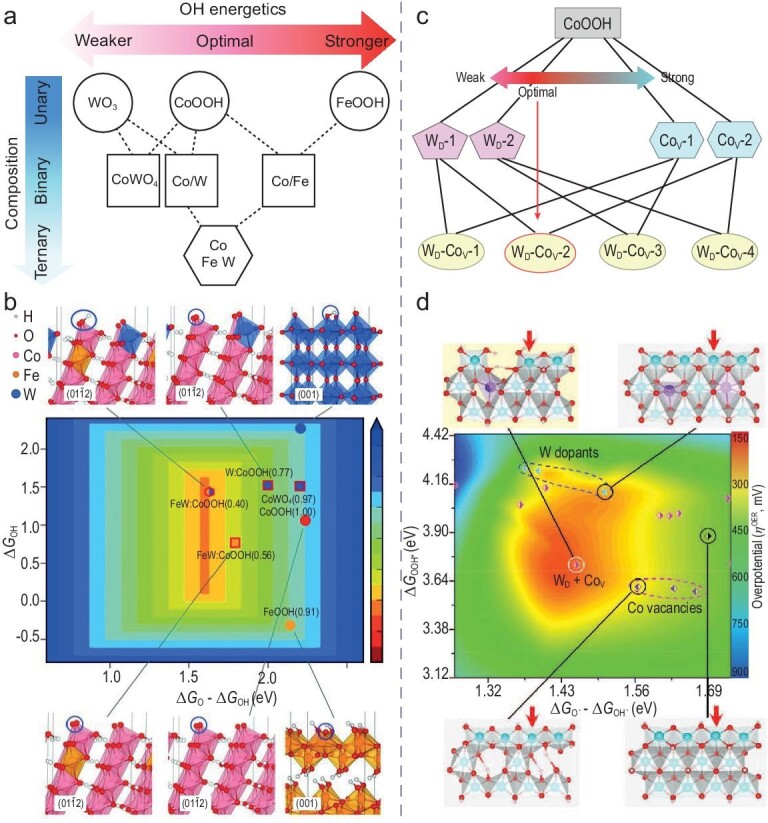
Application of interpolation principles for optimal doping scheme selection. (a) Change in OH adsorption energetics (Δ*G*_OH_) as a function of the increasing composition obtained by interpolation between the calculated pure phases: WO_3_ (001), CoOOH (01–12), FeOOH (010), and CoWO_4_ (010). (b) The corresponding OER activities calculated with DFT + U. Adapted with permission [8]. Copyright 2016, American Association for the Advancement of Science. (c) Design concept using the interpolation between W dopant and Co vacancy with opposite modulation effects in tuning the intermediate energetics for enhanced OER activity. (d) Contour plot of the theoretical overpotential as functions of Δ*G*_O*_ − Δ*G*_OH*_ and Δ*G*_OOH*_, Adapted with permission [75]. Copyright 2021, Wiley-VCH GmbH.

Moreover, the precise selection of suitable ligand metals and the meticulous adjustment of the electronic and geometric configurations of these atoms are crucial for initiating the OPM [37,76,77]. However, identifying the optimal single element or element combination from the periodic table poses significant challenges, especially using conventional experimental trial-and-error methods. Sargent has introduced a sophisticated theoretical screening methodology based on interpolation principles, using OH energetics as a predictive activity descriptor [8]. This method suggests that averaging elements with either excessive or insufficient OH adsorption tendencies could lead to desirable OH energetics (Fig. 4a). It assumes that the synthesis strategy ensures thorough mixing of the metals to prevent segregation into separate, inert metal oxide phases. In this regard, tungsten (W) is noteworthy for its ability to adeptly alter the valence state of 3d metal oxides, providing near-ideal adsorption energy for OER intermediates. The G-FeCoW catalyst, featuring high-valence tungsten, exhibited the lowest overpotential at the time (*E*_10_ = 191 mV) and maintained remarkable stability for over 500 hours. Its performance is ascribed to the intricate modulation of the 3d-CoFe oxidohydride by proton migration to the oxygen at the Co site (Fig. 4b), which triggers an OPM mechanism that aligns with the peak of OER energetics. Further investigations have expanded the concept of interpolation endpoints, demonstrating that they can include not only solid elements but also metal vacancies as a feasible alternative [75]. For example, Zhao *et al.* explored the synergistic effects between tungsten (W) dopants and cobalt vacancies (Co_v_) in CoOOH, which led to improved OER catalysis (Fig. 4c). The introduction of W and Co_v_ exhibits contrasting influences: W doping shifts the d-band center of CoOOH further from the Fermi level, whereas Co_v_ shifts it closer, resulting in lower and higher binding energies for OER intermediates, respectively. Consequently, W and Co_v_ work in concert to fine-tune the electronic state of the Co sites to an optimal intermediate binding energy, thereby significantly boosting catalytic performance (Fig. 4d).

In contrast to the M_1_–M_1_ type homonuclear dual-site configuration, the incorporation of the homogeneous diatomic M_2_–M_2_ configuration into the lattice matrix of M_1_ as active sites and redox centers yields distinct electronic and spatial alignments from the two conventional oxide structures (e.g. M_1_’s oxides and M_2_’s oxides), which potentially facilitates a more favorable direct O–O coupling (Fig. 2b) [42,71].

**Figure 5.**
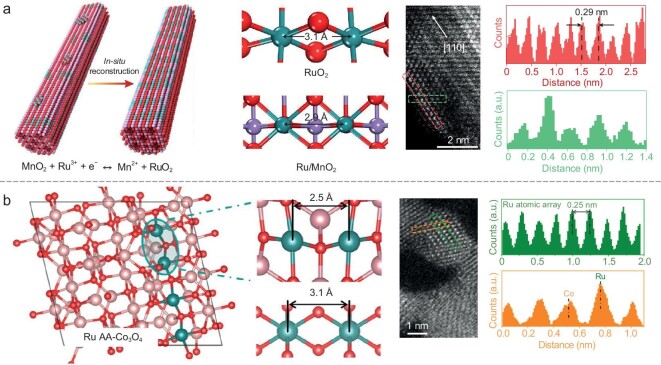
The catalytic center with an M_2_–M_2_ configuration. (a) *In-situ* reconstructed Ru atom array on α-MnO_2_ with enhanced performance for acidic water oxidation, simulated and experimental atomic images. Adapted with permission [20]. Copyright 2021, Springer Nature. (b) Oxygen radical coupling on short-range ordered Ru atom arrays, simulated and experimental atomic images. Adapted with permission [42]. Copyright 2024, American Chemical Society.

Lee and colleagues masterfully crafted Ru atom arrays on the α-MnO_2_ support (Ru/MnO_2_), where Ru atoms are strategically positioned to emulate the periodicity of Mn sites [20]. The Ru–Ru interatomic spacing, which achieved 2.9 Å and is narrower than that found in RuO_2_ (3.1 Å), is advantageous for the O–O radical coupling (Fig. 5a). The dynamic cation exchange that occurs between Ru ions and MnO_2_ during OER is crucial, as it transforms Ru atoms from small clusters into expansive arrays. In the fresh catalysts, ruthenium oxide nanoclusters dissolve and are redeposited onto MnO_2_, accompanied by the partial leaching of Mn. The restructured catalyst thus activates the OPM pathway, leading to enhanced OER performance, achieving 10 mA cm^−2^ at a potential of 161 mV vs RHE. Nonetheless, the stability of Ru/MnO_2_, which maintains 10 mA cm^−2^ for 200 hours, warrants further enhancement.

However, the stability of Ru/MnO_2_, which operates at 10 mA cm^−2^ for 200 hours, can still be improved. Building on this concept, Lu and associates synthesized Ru atom arrays on Co_3_O_4_ (Ru array-Co_3_O_4_) through cationic substitution (Fig. 5b) [42]. The Ru array-Co_3_O_4_ features symmetric Ru–O–Ru motifs with shorter Ru–Ru and longer Ru–O bond lengths compared to RuO_2_ and Ru/MnO_2_, which are thermodynamically more conducive for OPM initiation and attenuate the Ru–O covalent bond strength, thereby reducing the activation and participation of lattice oxygen. As a result, both oxygen vacancy formation and Ru dissolution are curtailed, with Ru array-Co_3_O_4_ demonstrating exceptional long-term stability over 1500 hours of testing and an ultra-low degradation rate of 0.07 mV h^−1^, alongside minimal ruthenium dissolution (0.066 μmol L^−1^). These two exemplary studies significantly advance the exploration of short-range ordered metal atom array electrocatalysts.

### Heteronuclear dual-atom sites configuration

OER electrocatalysts with heteronuclear dual-atom sites configuration (M_1_–M_2_) offer a strategy to optimize performance through precise tuning of catalyst composition (Fig. 2c) [71]. However, for catalytic reactions involving proton-coupled electron transfer (such as OER), the high surface energies of heterogeneous dual-atom catalysts and the low atomic utilization of crystalline bulk catalysts pose challenges in balancing OER activity and stability [16]. Therefore, it is necessary to adjust the electron transfer and spin polarization behaviors between M_1_ and M_2_, enabling the construction of synergistic catalytic center pairs that meet the spatial and electronic configuration requirements of the OPM.

**Figure 6.**
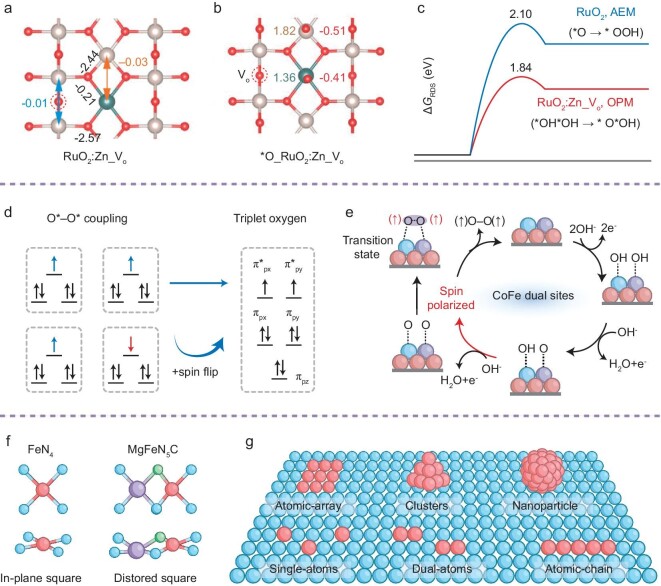
The catalytic center with an M_1_–M_2_ configuration. (a) Integrated crystal orbital Hamilton population (ICOHP) analysis of Ru–O, Ru···Ru, Ru···Zn, and Zn–O on the surfaces of RuO_2_: Zn_V_O_. (b) Bader charges analysis for Ru (brown), Zn (dark cyan), and O (red) sites on the double *O adsorbed surfaces of RuO_2_: Zn_V_O_. (c) The calculated energy profiles for the RDS of the corresponding reaction pathways, including the screened AEM for RuO_2_ and the OPM pathway for RuO_2_:Zn_V_O_. Adapted with permission [10]. Copyright 2023, Springer Nature. (d) The parallel arrangement of spin electrons in the oxygen–oxygen coupling of adjacent metal sites facilitates triplet oxygen production. (e) The schematic diagram of the CoFe dual sites for coupled O–O bonding during the OER (blue for Co atoms, purple for Fe atoms, red for S atoms). Adapted with permission [16]. Copyright 2024, Springer Nature. (f) The local geometrical configurations of Mg/Fe dual-atom electrocatalysts. Adapted with permission [19]. Copyright 2023, Wiley-VCH GmbH. (g) Schematic representation of the design of the dual-site electrocatalysts by the meticulous assembly of miniaturized functional atoms onto a substrate surface, including single atoms, dual atoms, atomic chains, arrays, clusters, and nanoparticles.

For instance, the oxidized state of Ru struggles to balance the activity and stability of OER electrocatalysts [20]. Specifically, due to the polarizing conditions of OER, Ru atoms undergo over-oxidation to produce water-soluble RuO_4_^2−^ species, resulting in poor electrocatalyst stability and performance degradation. Introducing electron-donating dopants into RuO_2_ can reduce the oxidation state of Ru and prevent its over-oxidation, but it may compromise the catalytic activity of OER [42]. This is because the strong binding of OER intermediates to the low-valence Ru sites hinders the deprotonation of the second water molecule necessary for the formation of the *OOH species. Lu and colleagues delicately modulated the electronic structure of the active Ru center by synergistic Zn doping and oxygen vacancies (Fig. 6a, b), which optimized the adsorption strength for oxygen intermediates [10]. Interestingly, they achieved modest adsorption of *OH at the Zn sites, establishing an alternative Ru–Zn pair oxidation pathway for the reaction. This optimized reaction path significantly reduced the energy barrier of the RDS (Fig. 6c) and inhibited the over-oxidation of the Ru active site, resulting in an ultra-low acidic OER overpotential of 173 mV vs RHE at 10 mA cm^−2^ and a stable operation of 1000 hours. Conversely, Zhang and colleagues demonstrated how to manipulate the adsorption and evolution of oxygen intermediates by adjusting the spin state of the active center during the OER process using a built-in electric field (Fig. 6d, e) [16]. They developed novel small-sized (1.5–3.0 nm) single-domain ferromagnetic CoFeSx nanoclusters that stimulate the presence of an internal electric field within the catalyst. In this setup, Co^3+^ (L.S, t_2g_^6^e_g_^0^) provides the strongest OH* adsorption energy, while Fe^3+^ (M.S, t_2g_^4^e_g_^1^) exhibits strong O* adsorption. The two-site synergistic Co–O–O–Fe intermediate induces spin-polarized water oxidation of key intermediate oxygen species and results in a spin-parallel alignment to lower the formation barrier of the O–O coupled intermediates, thereby facilitating the direct desorption of triplet oxygen (↑O = O↑). In a serendipitous discovery, Hou and colleagues ingeniously manipulated Fe into low spin states within Mg–Fe–N–C electrocatalysts by exploiting localized crystal field distortions, which arose from the substantial ionic radius disparity between Fe and Mg (Fig. 6f) [19]. This adjustment finely tunes the adsorption energy of oxygen intermediates on the Fe sites, while the Mg sites exhibit analogous bonding interactions with these intermediates, leading to a dual-atom adsorption framework that is conducive to the OPM mechanism. This metal-nitrogen-carbon construct presents a novel paradigm for designing bifunctional active sites by strategically modifying the catalyst surface. This approach departs from conventional bulk doping techniques and instead employs the precise integration of diminutive functional entities—ranging from single atoms to diatoms, atomic arrays, clusters, and nanoparticles—onto the substrate surface (Fig. 6g) [31,47,78,79]. These tailored configurations not only enhance the utilization of active sites but also improve charge transport and electronic structure, reduce internal resistance, bolster stability, and refine the interaction between the catalyst and the electrolyte [80]. The culmination of these advancements leads to a substantial boost in OER electrocatalytic activity. Particularly noteworthy are single atoms, diatoms, and atomic arrays, which due to their full atom utilization and modifiable unsaturated coordination environments, have attracted considerable interest, setting them apart from bulkier three-dimensional clusters and nanoparticles [43,81].

**Figure 7.**
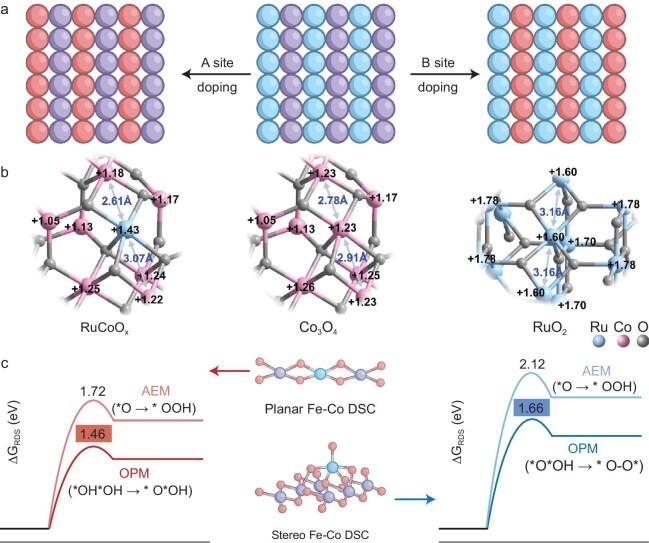
Strategic prioritization of doping positions. (a) Schematic representation of the doping positions within the active unit. (b) Electronic configurations of RuCoO*_x_* (left), Co_3_O_4_ (middle), and RuO_2_ (right) obtained based on Bader charge distribution calculations. Adapted with permission [38]. Copyright 2023, American Chemical Society. (c) Simulation models of planar and stereo Fe–Co dual-site catalysts and their calculated energy profiles of the RDS of the corresponding reaction pathways, including possible AEM and OPM pathways. Blue: Fe; purple: Co; red: O. Adapted with permission [9]. Copyright 2024, PNAS.

In the quest for ideal OPM-based OER electrocatalysts, selecting an optimal combination of elements is just the initial step. Equally crucial is the precise determination of the doping positions within the active unit, particularly for heterogeneous diatomic active centers (Fig. 7a). In crystal structures that feature multiple coordination environments, such as the tetrahedral (M_T_) and octahedral (M_O_) sites found in typical spinel structures, Xu and colleagues have shown that OER activity is predominantly influenced by the covalent interplay between these sites, creating an asymmetric M_T_–O–M_O_ framework [82]. Within this framework, the bond characterized by weaker metal-oxygen covalency dictates the exposure of the cationic sites and, consequently, catalytic activity. Here, the position of the second atom directly influences its electronic and geometric configurations, thereby shaping the reaction pathway and catalytic performance. Liang *et al.* have developed modified Co_3_O_4_ spinel electrocatalysts (RuCoO*_x_*), where Ru atoms substitute Co at the octahedral sites (Ru_oct_), forming highly symmetric Ru_oct_–O–Co_oct_ co-ligands with robust electronic coupling effects that significantly reduce the spatial separation between Ru and Co atoms, favoring direct dioxygen radical coupling (Fig. 7b) [38]. Notably, the integrity of the Ru_oct_–O–Co_oct_ synergistic coordination is preserved during acidic OER, facilitated by electron capture from the electron-rich tetrahedral Co (Co_tet_) atoms through bridging oxygen bonds.

Moreover, the relative positioning of heterogeneous atoms to the substrate—whether anchored or adsorbed—plays a decisive role in shaping the catalytic behavior and reaction pathways [83]. Anchored dopant atoms offer more stable reaction centers and mitigate active site loss, while adsorbed dopant atoms may increase the density of reactive sites and enhance reaction kinetics [84]. Wang and colleagues meticulously synthesized two catalysts with distinct spatial configurations: the planar Fe–Co dual-site catalysts (DSC), where Fe single atoms occupy the original Co sites in CoOOH (representing anchored atoms), and the stereo Fe–Co DSC, where Fe single atoms form a Fe–O–Co chelate structure with CoOOH via bridging oxygen (representing adsorbed atoms) (Fig. 7c) [9]. Planar Fe–Co DSC exhibits superior OER properties than stereo Fe–Co DSC. Comprehensive theoretical insights and experimental data demonstrated that oxygen intermediates are directly coupled to form *O–O* rather than *OOH by both configurations, thus bypassing the scaling relationship constraints of the conventional AEM pathway. More importantly, the ideal Fe–Co spacing and steric direction of the planar configuration are more conducive to dual-site cooperation, facilitating the dehydrogenation of the catalytic intermediates and the coupling for oxygen release, as compared to the stereo configuration. Consequently, the reaction kinetics were significantly accelerated, with the Gibbs free energy of the RDS decreasing from 1.66 eV (from *O*OH to *O–O*) for the stereo structure to 1.46 eV (from *OH*OH to *O*OH) for the planar structure. Interestingly, selectively anchoring Ru single atoms within the lattice, rather than on the surface, can establish a proton donor–acceptor pair, enabling oxygen intermediates to synergistically react at two distinct active sites—Ru_anc_ (proton donor) and bridging oxygen (proton acceptor), embodying the so-called proton donor–acceptor mechanism (PDAM) [74]. This approach offers a promising avenue to transcend traditional limitations. Conversely, Ru_ads_ atoms adsorbed on the carrier surface tend to follow an OER mechanism dominated by the LOM.

## IDENTIFICATION OF TRUE ACTIVE SITES AND REACTION PATHWAYS

The complex dynamic reconstruction and evolution of intermediates during reactions often obscure the actual reaction pathway [85]. However, the rapid advancement of nanoscale, high spatial-time-resolved complementary-*operando* techniques, and theoretical computational simulation is shedding light on these processes.

### High spatial-resolved atomic visualization techniques

**Figure 8.**
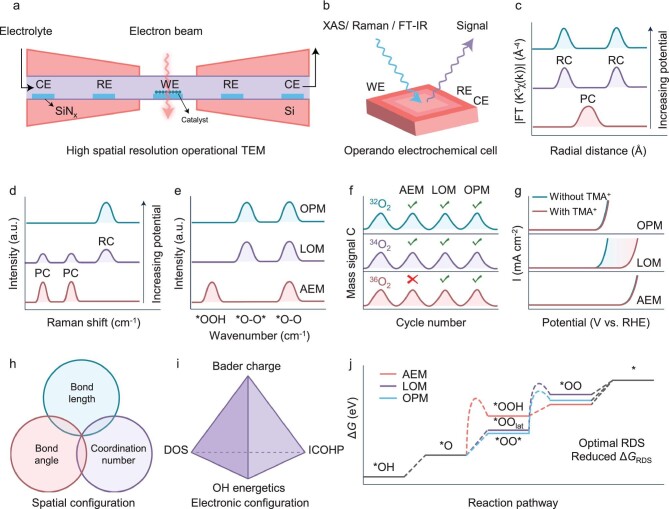
Identification of true active sites and reaction pathways. (a) Schematic representation of the high spatial resolution operational TEM with WE, RE, and CE representing the working electrode, reference electrode, and counter electrode, respectively. Adapted with permission [86]. Copyright 2019, American Chemical Society. (b) Schematic representation of the operational electrochemical cell represented by XAS, Raman, and FT-IR. (c) Schematic representation of the operational XAS, monitoring the transition of the coordination structure from pre-catalyst (PC) to real catalyst (RC) during the OER process in real-time. (d) Schematic representation of the operational Raman. (e) Schematic representation of the operational FT-IR. (f) Illustrative diagram of product information corresponding to three reaction paths by the ^18^O isotope labeling technique combined with DEMS. (g) Illustrative diagram of the chemical probe (TMA^+^) effect on electrocatalysts driven by different reaction pathways. (h) Illustrative diagrams of simulated spatial (h) and electronic configurations (i) of the target catalysts by theoretical calculations. (j) Density functional theory (DFT) based on first principles is utilized to simulate the Gibbs free energy of the reaction.

Direct visualization of local interfacial evolution at fine spatial scales is crucial for the definitive pinpointing of potential active sites within composite structures [87]. Aberration-corrected scanning electron microscopy (AC-STEM) has recently made atomic-level visualization possible, enabling the design of precisely defined model catalysts [88]. Moreover, vibrational electron energy loss spectroscopy (EELS) within STEM emerges as a potent technique for investigating the vibrational properties of materials with high spatial resolution [89]. The electrified solid-liquid interfaces are pivotal in various electrochemical processes, where electron and mass transport can lead to structural changes that significantly impact reaction pathways. The surface restructuring of electrocatalysts during the OER process can profoundly influence catalytic mechanisms. Thus, directly probing the atomic dynamics at solid-liquid interfaces under electrical bias is critically important but remains challenging due to the interfaces being submerged in liquid electrolytes and the limited spatial resolution of existing methods. Fortunately, the advent of cutting-edge *in situ* electrochemical cell transmission electron microscopy (EC-TEM) now permits the direct observation and analysis of charged solid-liquid interfaces in their native environment, bypassing the need for freezing or drying. This innovation paves the way for real-time atomic-scale visualization of complex electrochemical reactions (Fig. 8a) [86,90–92]. Leveraging *in situ* EC-TEM and EELS, Yan *et al.* provided the first detailed account of lattice sulfur atoms in NiCo-based sulfides being partially substituted by oxygen from the electrolyte, a process initiated by pre-catalytic voltage and hydroxyl molecules in the solution, leading to the emergence of lattice oxygen-sulfur coexisting shell surfaces [93]. This S–O exchange is facilitated by subtle shifts in metal-sulfur coordination, influenced by specific nickel and cobalt occupancies, which in turn lowers the energy barriers for surface reconstruction. More recently, Zheng *et al.* introduced an advanced polymer electrochemical liquid cell for transmission electron microscopy (TEM), enabling direct observation of atomic dynamics at electrified solid-liquid interfaces during electrochemical reactions. This *in situ* imaging technique through liquids offers new avenues to investigate atomic dynamics on OPM-based electrocatalysts, and ascertain the influential factors crucial for promoting the OPM mechanism during OER [92].

### High time-resolved *in-situ* spectroscopic techniques

*Operando* spectroscopic techniques represented by X-ray absorption spectroscopy (XAS), Raman spectroscopy, and Fourier transform infrared spectroscopy (FT-IR) provide a powerful, non-destructive tool for detecting the time-resolved dynamic response of active sites and reaction intermediates (Fig. 8b) [3,89,94]. In the typical MX pre-catalyst (where M represents transition metal elements and X represents non-metallic elements), the disappearance of MX and the concurrent enhancement of MOOH at progressively increasing applied bias were observed using *in situ* X-ray absorption spectroscopy and Raman spectroscopy (Fig. 8c, d) [16,31,91]. Moreover, the characteristic differences in the formation mode of oxygen molecules suggest that the reaction pathways can be deduced from the evolution of oxygen intermediates, detectable by *in situ* FT-IR [95]. The simultaneous presence of *OOH and *O–O indicates AEM, while the coexistence of *O–O* and *O–O suggests potential LOM and OPM (Fig. 8e) [20,41,42]. For example, the peaks at 1110 and 1070 cm^−1^ identified in the Ru array-Co_3_O_4_ electrocatalyst during OER, which intensified as the potential increased during operational FT-IR, are ascribed to the linearly coordinated metal–O–O intermediates (Ru–O*–O*) and the oxygen bridges between two neighboring Ru atoms (Ru–O*–O*–Ru), respectively. These spectral features, recognized as the O–O stretching vibrations of adsorbed dioxygen species, suggest the operation of the OPM. This is in contrast to RuO_2_ or other Ru-based electrocatalysts, where the AEM typically involves Ru–OOH* intermediates [42].

### ^18^O isotope labelling techniques

By coupling ^18^O isotope labeling with differential electrochemical mass spectrometry (DEMS), we can detect reaction products to elucidate the pathways involved [18,96]. Initially, a freshly prepared electrode was cycled through continuous cyclic voltammetry (CV), typically for tens of cycles between 1.1 and 1.7 V vs RHE, in an electrolyte using H_2_^18^O as the solvent [20]. This step was to maximize the ^18^O labeling on the catalytic surface. The ^18^O-labeled electrode was then thoroughly rinsed with deionized water to eliminate any physically adsorbed H_2_^18^O from the catalyst surface, followed by use in an electrolyte containing H_2_^16^O. Next, the O_2_ gas products evolved by the ^18^O-labeled catalyst during OER were characterized using DEMS. Ordinarily, during the labeling process, ^18^O may be incorporated into both the lattice oxygen sites and surface-adsorbed oxygen sites. Hence, for the AEM mechanism, one would detect the products ^32^O_2_ and ^34^O_2_, but not ^36^O_2_. In contrast, for the LOM and OPM mechanisms, all three isotopic oxygen molecules—^32^O_2_, ^34^O_2_, and ^36^O_2_—could be observed (Fig. 8f) [9,20]. To effectively distinguish between LOM and OPM, specific chemical probes such as tetramethylammonium cation (TMA^+^) are often employed. The O_2_^2−^ species produced during LOM can strongly interact with TMA^+^, significantly diminishing activity, whereas TMA^+^ does not impact the AEM and OPM processes in the same manner (Fig. 8g) [96,97].

### Theoretical computational simulation

Theoretical computational simulation has become an indispensable instrument in the realm of modern catalysis [15]. Critical geometric parameters, including bond lengths, bond angles, and coordination numbers, can be deduced from the spatial arrangements of atoms (Fig. 8h) [42,49]. Moreover, comprehensive analyses of Bader charge, density of states (DOS), integrated crystal orbital Hamilton population (ICOHP), and hydroxide (OH) adsorption energy can proficiently delineate the electron transfer dynamics and intermediate adsorption states of catalysts, thereby probing the electronic structure (Fig. 8i) [31,38,41,98]. These simulated spatial and electronic configurations facilitate the strategic identification of ideal OPM-based OER electrocatalysts from both known and novel materials [48]. Additionally, density functional theory (DFT) grounded in first-principles calculations provides insights into the Gibbs free energy of reactions, uncovering the rate-determining steps (RDS) in complex reaction pathways. This information is pivotal for targeted material refinement and elucidating the mechanisms underlying exceptional OER performance (Fig. 8j) [20,42,89]. For example, the potential OER mechanisms for the electrocatalyst Ir–SrMnO_3_ were explored using DFT calculations, which indicated that Ir–Mn dual atoms with optimal spatial and electronic configurations could activate the OPM, presenting the lowest energy barriers in comparison to AEM and LOM. This suggests that oxygen molecules could be efficiently generated through direct O–O coupling via the OPM [99].

## SUMMARY AND OUTLOOK

**Figure 9.**
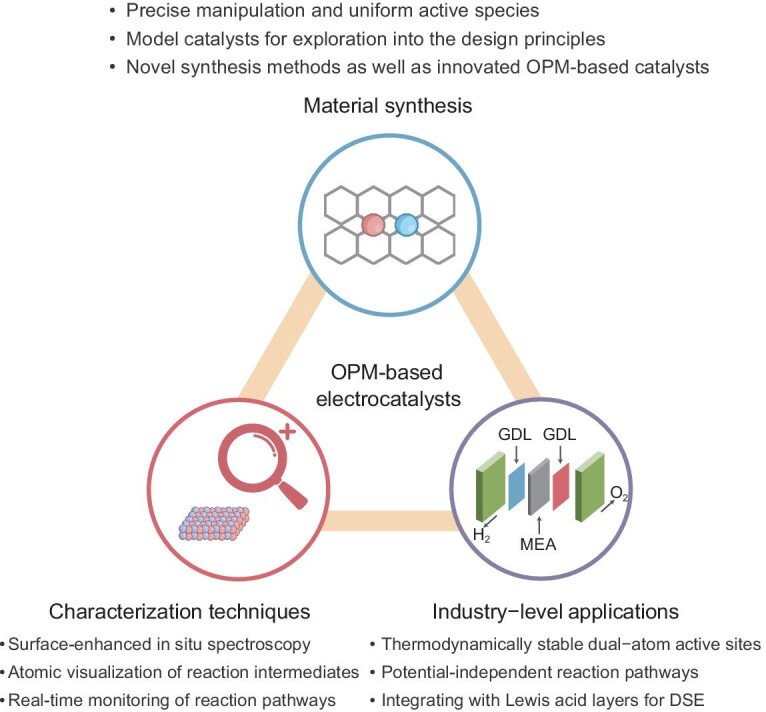
The remaining challenges and opportunities for OPM-based electrocatalysts.

The pursuit of superior OER electrocatalysts has driven us to explore the intricate structure–activity–stability relationship, with the OPM pathway heralded as a potential solution to overcome the inherent limitations of previous AEM and LOM pathways without compromising activity. This review encapsulates the principles of OPM, design criteria, strategies, and cutting-edge methodologies for pathway identification. Despite significant strides, the journey ahead for OPM-based OER electrocatalysts is rife with challenges and ripe with opportunities (Fig. 9).

### Precise and uniform synthesis of OPM-based electrocatalysts

As previously discussed, a variety of synthesis methods have been developed to produce OPM-based electrocatalysts. However, the approaches reported are specific and not readily adaptable to other materials. A significant challenge remains in achieving a structure where one metal atom is exclusively bonded to another, and in uniformly synthesizing active sites in the form of dimers, which initiate the OPM for OER. The difficulty in forming precise configurations of dual-atom sites arises when isolated single atoms or metal nanoparticles inadvertently emerge due to the use of large or small amounts of metal precursors [71]. This non-uniformity in active sites hinders the full exploitation of OPM's potential. Additionally, the mixed mechanisms complicate accurate evaluations of the relationship between structure and performance. Furthermore, even when the amount of doping metal precursors is precisely controlled and dual-atom pairs are fabricated, they may not be suitable for the OPM due to inappropriate distances or angles caused by unsuitable doping elements. Current synthesis methods for dual single-atom catalysts, such as high-temperature pyrolysis, wet impregnation, and atomic layer deposition, rely on confinement or molecular anchoring effects [40]. However, these methods’ limited ‘precise engineering’ capabilities lead to structural inhomogeneities, resulting in catalysts that are often a mixture of single atoms, dual atoms, or cluster sites. Therefore, there is a pressing need for novel OPM-based electrocatalysts and their synthesis methods, and an exploration into the principles of designing OPM-based electrocatalysts is highly desirable. Single-atom catalysts supported by carbon-based materials provide ideal platforms for studying fundamental catalytic mechanisms and contribute to the rational design of catalysts with tailored activity for specific reactions. Thus, dual-atom active sites on carbon-based supports may offer an ideal platform for investigating OPM-related pathways and principles. Consequently, refined synthetic approaches, guided by theoretical computational simulations, can enable the precise and scalable construction of targeted catalysts, which will further elucidate the complex interplay between OER catalytic kinetics and structure in these well-defined catalyst models.

### From lab-scale research to potential industry-level applications

Although OPM-based electrocatalysts show great potential for high activity and stability, the evaluation remains stuck in laboratory conditions. It is crucial to conduct activity and stability assessments at industrial current densities (200 mA cm^−2^) to determine their viability for large-scale electrolyzer deployment. In general, there are three arduous challenges that should be addressed for commercial applications: (1) unlike laboratory conditions (25°C, 1 M KOH), industrial applications typically employ a higher temperature and concentration of the electrolyte (usually 80°C, 6 M KOH) [100]. This is because an increased electrolyte concentration boosts ionic strength and conductivity, while a higher temperature enhances diffusion kinetics. However, the elevated concentration of KOH heightens the vulnerability of catalysts to corrosion and degradation, a situation that is further aggravated at 80°C [101]. These factors can challenge the stability in industrial settings, potentially leading to deactivation due to the destruction of the dual-atom active sites that are critical for the OPM mechanism. (2) Bubble jamming during the OER process is a significant issue that greatly hampers mass transfer efficiency and obscures active sites [102]. This problem is exacerbated at high current densities during industrial-scale electrolysis, potentially accounting for a substantial fraction of total energy losses. Additionally, the concentration polarization and intense localized stresses caused by large bubble formation can negatively influence electrode stability and hasten the degradation of the active structure, ultimately leading to inactivity [103]. Addressing these challenges is crucial for all OER electrocatalysts, especially for OPM-based catalysts that are poised to deliver top-tier performance. While the development of microstructured porous electrodes with enhanced gas evacuation capabilities can improve the directional transport of gas bubbles, the volume of bubbles increases with rising current densities, making timely evacuation difficult and potentially damaging the delicate porous nanostructures on the catalyst. Consequently, there is a pressing need to devise more effective strategies for efficient bubble evacuation at industrially relevant high current densities, which will enhance both the activity and stability for commercial applications. (3) Both experimental and theoretical studies have suggested that the reaction pathway may be potential-dependent, potentially shifting from OPM to LOM or AEM at elevated potentials [12,104]. To meet industrial catalysis requirements, maintaining the stability of OPM-based electrocatalysts for several months is crucial to avoid economic losses from frequent catalyst replacement. Therefore, there is a pressing need to develop ingenious synthesis strategies to construct precise dual-atom active sites with long-term thermodynamic stability for OPM, even under elevated potentials. Significant efforts at the atomic/molecular level and in understanding the local coordination structure are essential to preserve dual-atom sites for OPM at high current densities, thereby maintaining the integrity of the OPM pathway.

Additionally, the reliance on large volumes of high-purity water for hydrogen production could exacerbate issues of water distribution inequality and resource scarcity. Direct seawater electrolysis (DSE), utilizing an abundant and natural electrolyte source, represents a more forward-looking approach to obtaining clean energy. Innovations in OPM-based catalyst design may be beneficial for suppressing unwanted side reactions under near-neutral conditions, especially in the presence of high chloride ion concentrations in seawater. A recent approach by Qiao *et al.*, involving the creation of a localized alkaline microenvironment through the introduction of a Lewis acid layer on the catalyst surface, has shown promise [60]. This method enhances activity while mitigating harmful chlorine chemistry and precipitate formation. This innovative concept could be extended to design advanced OPM-based catalysts in the future, opening an avenue for direct seawater electrolysis.

### Concise and precise characterization techniques

Although the direct O–O coupling for oxygen evolution has long been proposed, the study of the OPM for OER is still in its nascent stage. Compared to AEM and LOM, there are more unknown factors that may affect the OPM pathway, and even the identification of the OPM itself remains a challenging task. Furthermore, the dynamic evolution of active sites, which should not be considered static, adds complexity to the study and exploration of mechanisms during OER. Therefore, the development of advanced characterization tools, complemented by theoretical calculations, to elucidate the true active sites and identify the reaction pathways—particularly for OPM-based electrocatalysts—is highly desirable. Generally, the recognition of reaction pathways requires the cross-corroboration of several tools—including, but not limited to, *in situ* FT-IR spectroscopy, DEMS, and TMA^+^ chemical probes—to gather comprehensive information. However, this approach often leads to low reliability and efficiency. Specifically, the weak vibrational intensity of *in situ* infrared spectroscopy typically results in several broad peaks that are difficult to distinguish, not to mention the challenging testing conditions. Thus, the vigorous development of surface-enhanced *in situ* electrochemical spectroscopy is a direction worth considering. Looking further ahead, real-time visualization of the evolution of reaction intermediates at the atomic level, although extremely demanding, will undoubtedly open up groundbreaking opportunities for identifying OPM-related reaction pathways. The vast parameter space inherent to the high-performance catalyst design presents a significant challenge in the development and optimization of OPM-based catalysts. The integration of machine learning (ML) within the material science domain has proven invaluable in extracting insights and discerning patterns from extensive datasets across various fields. The growing prominence of ML is instrumental in expediting the exploration and discovery of heterogeneous catalysts, enabling a more effective navigation through the complex parameter space and contributing to a deeper understanding of material properties and behaviors—an essential aspect of ‘Industry 4.0’ [105,106]. The activity descriptors in catalysis can establish correlations between the physicochemical properties of catalysts and their performance, significantly reducing time and cost in the design and development of efficient electrocatalysts for OER. While numerous descriptors for the OER activity have been proposed, precisely tailored activity descriptors for OPM-based electrocatalysts remain elusive. Leveraging ML to identify better-suited activity descriptors with refined independence and controllability for OPM-based electrocatalysts could drastically cut down on the time and expense associated with the trial-and-error approach in the OPM-based catalyst development, representing a highly significant yet challenging scientific endeavor [70].
